# Gastrointestinal Disturbances Associated with the Consumption of Sugar Alcohols with Special Consideration of Xylitol: Scientific Review and Instructions for Dentists and Other Health-Care Professionals

**DOI:** 10.1155/2016/5967907

**Published:** 2016-10-20

**Authors:** Kauko K. Mäkinen

**Affiliations:** Institute of Dentistry, University of Turku, Lemminkäisenkatu 2, 20520 Turku, Finland

## Abstract

Sugar alcohols (polyols) are used in food manufacturing and in medical tests and examinations. d-Glucitol (sorbitol) and d-mannitol were previously the most common alditols used for these purposes. After the 1960s, xylitol became a common ingredient in noncariogenic confectioneries, oral hygiene products, and diabetic food. Erythritol, a polyol of the tetritol type, can be regarded as the sweetener of the “next generation.” The disaccharide polyols maltitol, lactitol, and isomalt have also been used in food manufacturing and in medical tests. Consumption of pentitol- and hexitol-type polyols and disaccharide polyols may cause gastrointestinal disturbances at least in unaccustomed subjects. The occurrence of disturbances depends on consumer properties and on the molecular size and configuration of the polyol molecule. Adaptation may take place as a result of enzyme induction in the intestinal flora. Some of the literature on xylitol has been difficult to access by health-care professionals and will be reviewed here. Research and clinical field experience have found no pathology in polyol-associated osmotic diarrhea—the intestinal mucosa having normal basic structure, except in extreme instances. Xylitol is better tolerated than hexitols or the disaccharide polyols. Erythritol, owing to its smaller molecular weight and configuration that differ from other alditols, normally avoids the gastrointestinal reactions encountered with other polyols. This review will also touch upon the FODMAPs diet concept.

## 1. Introduction

The use of sugar alcohols (polyols) in the manufacturing of foods, medicines, and oral hygiene products has increased considerably during the past decades. Examples of more frequently used polyols include simple alditols such as erythritol, xylitol, d-glucitol (sorbitol), and d-mannitol and disaccharide sugar alcohols such as maltitol, lactitol, and isomalt. Sugar alcohols have been used in surprisingly numerous medical, cosmetic, techno-chemical, and similar applications. Xylitol-based infusion therapy currently comprises one of the largest single applications of this alditol [1]. Xylitol-containing chewing gums have also been employed in various medical studies related to cognitive function, mastication, drug delivery, physiologic tests, and others [2].

Xylitol and d-glucitol are used in chewing gums and troches aimed at reducing the incidence of dental caries [2]. Physiologically and physicochemically, these substances are normally absorbed slowly from the intestinal lumen and may cause so-called osmotic diarrhea in some individuals if the amounts consumed are too high [3]. Such symptoms may occur especially in subjects unaccustomed to sugar alcohols, as has been found already since 1960s [3–9]. The occurrence of diarrhea, however, depends on a multitude of factors such as the person's weight, the composition and structure of the rest of the simultaneously consumed diet [10, 11], state of fasting, the type of food that contains the sugar alcohols (liquid and solid consumables normally have different effects) [8], and other factors [4]. What is even more decisive is the molecular structure of the ingested sugar alcohol. The size and symmetry of the sugar alcohol molecule, and the number of hydroxyl groups present in the molecule, significantly influence the behavior of each sugar alcohol along the entire length of the alimentary tract. It is possible that these causative factors are not known to all health-care workers.

Despite the many positive effects of sugar alcohols, their consumption is frequently linked also to irritable bowel syndrome (IBS) and abnormal flatulence [9, 12], which affect quality of life negatively and result in a considerable economic burden in terms of health-care costs. However, it has become evident that sugar alcohols can be beneficial in the treatment of chronic constipation [13]; for example, d-glucitol has been exploited in several commercial preparations. Therefore, dentists, physicians, and other health-care workers should be made to understand where to focus their attention when communicating with patients, detecting false opinions and misconceptions about diarrhea, flatulence, IBS, and constipation, and correcting them on the basis of scientific evidence and long-term clinical experience. Tuck et al. [12], Xiao et al. [14], and other authors have provided information on the gut function-enhancing effects of “indigestible sugars” (i.e., sugar alcohols and certain oligosaccharides) [3, 5, 6, 15–21]. These aspects are represented in this review by older German publications [3, 19] and those from the former Soviet Union [13] (*vide infra*).

No previous dental article has specifically focused on osmotic diarrhea associated with excessive consumption of sugar alcohols. Consequently, the primary objective of this article is to review the gastrointestinal research carried out with sugar alcohols with special consideration of simple dietary alditols which have been used principally for dental purposes. Because xylitol has been used for decades in consumer products favored by children—in ever-increasing amounts—this review will especially focus on the experience gathered with xylitol in clinical feeding studies. Some of the older scientific literature on xylitol has been difficult to access by regular health-care workers and will be reviewed here. Erythritol, “the next-generation” sweetener of a tetritol nature, will be concisely discussed owing to its generally recognized gastrointestinal safety [15]. The newly developed FODMAPs concept will also be touched upon. It is necessary to emphasize that osmotic diarrhea occasioned by excessive consumption of carbohydrates and polyols is not a disease, but rather a simple physicochemical response of the intestinal tract to the presence of slowly absorbed carbohydrates or polyols in the gut lumen.

## 2. Physicochemical Considerations and Introduction of the FODMAPs Concept

In the systematic chemical nomenclature sorbitol is called d-glucitol. This term will be used in the present text since the presence of this molecule in the structure of certain disaccharide sugar alcohols is most appropriately depicted using the official chemical term. The symmetric xylitol molecule should not be written with either the d or l prefix. For* meso*-erythritol or* i*-erythritol, the simple term erythritol will be used. Mannitol requires a d or l. For the present purpose, glucose, galactose, and fructose are shown without specifying the configuration (i.e., d), unless the names appear in those of disaccharide sugar alcohols.

When a person consumes solutions containing excessive amounts of carbohydrates and polyols (or salt), water can draw from the body into the gut lumen, causing osmotic diarrhea. This can naturally also result from a disease condition (such as pancreatic disease). Although the present review will focus only on cases with osmotic diarrhea that may occur in healthy subjects who consume excessive quantities of sugar alcohols, it is necessary to recall that acute osmotic effects may also result from consuming too great a quantity of processed grains and cereals [16] and certain fruits and vegetables [17] (*vide infra*, Section 3). In healthy individuals, too-large quantities of common substances such as vitamin C, magnesium salts, lactose, and certain antibiotics may cause severe cases of osmotic diarrhea and bowel distension. Owing to its simple physical cause, osmotic diarrhea normally stops completely when the use of the offending agent is discontinued.

Experiments involving oral administration of sugar alcohols have normally been carried out using glucose or fructose as comparisons. It has been found that, in most subjects, glucose has no laxative effect even in extraordinarily high dosages. For fructose, the threshold of a single dose is normally around 70–100 g. Fructose represents an important point of comparison, since consumers' own judgments as to the origin of osmotic diarrhea following consumption of sugar alcohols are often confused by simultaneous consumption of fructose. The role of fructose and d-glucitol in the etiology of IBS has been somewhat controversial when these substances are ingested together [18]. It nevertheless appears that the degree of symptom provocation is related to the amounts present in such a mixture but may not be related directly to the extent of colonic hydrogen production [18].

Sugar alcohols behave in the gut lumen in different ways, and their effects are not identical. Sugar alcohol molecules react in the gut lumen as physical and chemical entities based on their molecular mass, number of hydroxyl groups present in the molecule, the spatial orientation of those groups, and the overall symmetry of the molecule. All common dietary alditols are characterized by the presence of only two types of chemical groups, that is, CHOH and CH_2_OH. The number of OH groups present in these molecules is shown in Table 1, which also reveals how the praxis of expressing concentrations differs significantly. These differences have also generated misunderstandings, since most clinical and nutritional reports customarily give the amount of sugar alcohols as percentages. The true chemical concentrations can be significantly different, however. The case of erythritol and sucrose serves as an example. The molarity of sucrose in a 10% (w/w) solution is only about one-third of the molarity of erythritol at the same 10% concentration. In physiological studies, it may be preferable to use chemical activities, that is, the chemical concentrations (molarities), to make the cases chemically comparable.

The intestinal absorption of xylitol is almost totally limited to the mechanism of permeation which applies to all strongly hydrophilic substances. The driving force behind free diffusion of xylitol is the direction of the concentration gradient between the intestinal lumen and the outside compartment [1, 3, 4, 8–14, 19]. These papers have concluded, among other things, that in case facilitated diffusion of xylitol is involved, the transport system must exert very low affinity to xylitol. In free diffusion, the uptake of the substance from the intestinal lumen takes place because of a simple physicochemical process through the hydrophobic pores in the membrane. In this process, molecular size is of particular significance. This parameter is to a certain extent indicated by the molecular weight of the substance. It is obvious that relatively extended molecules are in themselves ill-suited to the permeation process.

The xylitol molecule is totally symmetrical and small compared with the d-glucitol molecule whose molar mass and dimensions are larger and which is also relatively asymmetrical (in the latter molecule, the hydroxyl groups on C4 and C5 are in the d-configuration). The molar mass and symmetry of d-mannitol also differ significantly from those of xylitol. Hence the consumption of d-glucitol and d-mannitol generates far more severe gastrointestinal disturbances than xylitol. A comparison between the molecular weights of xylitol (152.1) and glucose (180.2) suggests that xylitol absorption amounts to approximately 50% of the free diffusion of glucose. In this comparison, the intestinal uptake of d-glucitol (182.2) may be about 60% of the absorption of xylitol.

While glucose is virtually completely absorbed in the upper part of the small intestine, xylitol is normally only partly absorbed in the upper part and is present in considerable amounts in the lower region of the small intestine. However, this depends on the quantity of xylitol consumed. Experience from the Finnish Turku Sugar Studies [4, 20] also indicated that xylitol-associated diarrhea can be prevented by simultaneous administration of bulky food. However, bulky food does not considerably increase the absorbability of xylitol, since the preventive effect results primarily from delayed emptying of the stomach. The presence of plant fibers may bind water, mitigating xylitol-associated diarrhea. As soon as the causative agent (xylitol) is removed, the tendency of osmotic diarrhea passes. Also, no irritation is generally observed in mucous membranes, except in extreme instances. Total absorption of xylitol and lessened osmotic diarrhea are more likely to occur when smaller quantities are consumed as part of a regular diet.

When xylitol is administered in an isolated form in beverages, the xylitol molecules are no longer sufficiently absorbed in the small intestine and will reach the colon. This concerns other pentitols and all hexitols as well. Therefore, consumption of polyol-containing beverages—apart from those based on erythritol—is not generally recommended. In the colon, bacterial action converts d-glucitol to low-molecular decomposition products with much higher osmotic potential than in the case of xylitol.

A form of adaptation to xylitol was first discovered in animal feeding studies and subsequently also in humans. Most notably this phenomenon was discovered in the two-year xylitol feeding study in Turku [4, 20]. This adjustment has normally been linked to an enzyme induction; the activity levels of liver sorbitol dehydrogenase which catalyzes the initial oxidation of xylitol increase during habitual consumption of xylitol.

The largest single boluses of sugar alcohols that can elicit osmotic diarrhea in adult subjects differ based on experimental details. Typical results obtained in feeding studies are shown in Table 2. Such values must not be regarded as universally valid. The true effects depend on circumstances; evaluations conducted by different research teams may not be exactly congruent.

Based on the interest focused on diets that reduce intake of poorly absorbed small-molecular-size carbohydrates, a particular FODMAP concept was developed [16, 17, 21–25]. FODMAPs are short-chain carbohydrates that are poorly absorbed in the small intestine. The term is an acronym derived from “Fermentable Oligo-, Di-, Mono-Saccharides, and Polyols.” The FODMAPs research and the low FODMAPs diet concept was developed at the Monash University in Melbourne. It is important to emphasize the role of polyols, such as xylitol, d-glucitol, and d-mannitol, in the FODMAP group of carbohydrates. Understanding the importance of dietary FODMAPs will be assisted by comprehensive food composition data [17]. Although sugar alcohols can be used to alleviate chronic constipation, it is thus obvious that sugar alcohols—with the exception of erythritol—should generally be avoided as part of low FODMAPs diet. Dental health professionals are encouraged to get familiar with the FODMAPs concept.

## 3. The Pivotal Role of ****d****-Glucitol and ****d****-Mannitol

Older literature is cited here on purpose in order to emphasize the existence of gradually growing clinical interest in this area of research. One of the earliest scientific reports on the very slow absorption rate of d-glucitol was published by Dahlqvist and Telenius [26]. Intractable diarrhea associated with the use of d-glucitol has later been frequently reported in the clinical literature. This has most often resulted from the use of d-glucitol as a sweetener and bulking agent in dietetic candies and chewing gum, although use of d-glucitol as a vehicle for suspending active drugs for oral preparations can also cause intractable diarrhea [27]. Only about 35 years ago several researchers seemed surprised by the appearance of “dietetic food diarrhea” caused by excessive d-glucitol and d-mannitol consumption [28] and by the metabolism of d-glucitol by gut bacteria [29], even though already in the late 1950s some physicians had noted the very slow absorption of d-glucitol from the small intestine [30]. The situation was partly a result of the limited information available in pediatric textbooks concerning diarrhea caused by poorly absorbed osmotically active substances. Pediatric gastroenterology texts contained only passing references to this form of diarrhea, caused by dietetic, d-glucitol-containing candies or chewing gum [31, 32]. A typical case report normally follows a series of events similar to the following example: A 3-year-old boy consumes six full packs of a d-glucitol chewing gum brand. The consumed amount of d-glucitol is about 40 g. About one hour after the gum ingestion the child complains of abdominal cramps and explosively passes about 500 mL of thin liquid stool.

Several case reports began to call attention to d-glucitol-containing “diet foods.” Special “pink” diarrhea was caused by d-glucitol-containing vitamin C supplements; the pink color was attributed to the cochineal dye added to the preparation [33]. (“Cochineal” originally referred to the red dye manufactured from the dried bodies of female cochineal insects or wood lice; the dye can also be synthesized.) As late as 1984, physicians alerted public health experts to the diarrheal potential of d-glucitol [34], as the quantities of d-glucitol used in the candy and food industry increased significantly. The popular use of d-glucitol in cough mixtures, cough drops, and various pharmaceutical syrups also began to receive attention; all of them have been reported as potential causes of diarrhea especially in infants. Such products were, however, beneficial from a dental standpoint, provided that d-glucitol replaced all fermentable carbohydrates previously used in such products. This was clearly a positive property of d-glucitol-containing items.

It is well known that d-glucitol and d-mannitol are present in a wide variety fruits and other plant material [16, 17]. The concentration of d-glucitol in dried fruit, such as prunes, may reach levels that can contribute to diarrhea. Anecdotal evidence suggests that, historically, pediatricians advised mothers to give prunes to children with constipation. Modern scientific research concerning the FODMAPs concept has, however, more quantitatively underlined the role of d-glucitol and d-mannitol in osmotic diarrhea and their occurrence in natural products [16, 17] (*vide supra*).

As stated above, IBS was also reported to result from the ingestion of mixtures of fructose and d-glucitol [18]. Yao et al. [9] concluded that increased and discordant absorption of d-mannitol and d-glucitol occurs in patients with IBS compared to that in healthy controls. Both alditols induced gastrointestinal symptoms in patients with IBS independently of their absorptive pathway, indicating that dietary restriction of the alditols may be efficacious. Xiao et al. [14] emphasized the health-enhancing properties of so-called indigestible sugars which include a large assembly of simple and complex dietary carbohydrates including monosaccharides, oligosaccharides, and certain alditols and disaccharide polyols. d-Glucitol, erythritol, and xylitol were presented as health-enhancing substances. Similar comments have been made elsewhere [35, 36], with evidence that particularly fructose conditions the gut microflora. “Toxicity” of d-mannitol and d-glucitol was discussed as early as 1941 [37]. Recent IBS papers included those of Shepherd et al. [38], Tuck et al. [12], Respondek et al. [39], Goebel-Stengel and Mönnikes [40], and El-Salhy [41].

Some researchers also interpreted other d-glucitol effects as positive: d-glucitol therapy reportedly improved psychomotor performance in cirrhotic patients [29]. Patients with hepatic encephalopathy improved in all five mental function tests, whereas similar patients not receiving d-glucitol showed no improvement. d-Glucitol has naturally been exploited in medical practice as a cathartic preparation, another useful sugar alcohol application. A comparison between d-glucitol and lactulose showed that both were extensively fermented by the colonic flora [42]. It was suggested that the much cheaper d-glucitol could be used in the treatment of postsystemic encephalopathy. The medical literature has, however, simultaneously been replete with case reports and clinical studies relating detrimental d-glucitol effects, that is, intolerance to this sugar alcohol, as evidenced by the literature references shown above. Based on field experience and clinical evaluations, most experts contend that d-glucitol may produce osmotic diarrhea if ingested in amounts of 20 g to 50 g. A debate on the possibility of glucose-stimulated influx of d-glucitol across the human jejunal mucosa has continued since the late 1990s [43].

## 4. Also Disaccharide Sugar Alcohols May Cause Osmotic Diarrhea

Maltitol (*O*-*α*-d-glucopyranosyl-1,4-d-glucitol; molar mass 344.31) is a disaccharide sugar alcohol derived from maltose by dehydrogenation. Owing to the hydrolytic cleavage of maltitol by intestinal enzymes, free glucose and free d-glucitol are formed. The liberated glucose molecules are absorbed virtually completely, whereas the liberated d-glucitol is incompletely absorbed, contributes to osmotic diarrhea, and is eventually subject to microbial fermentation in the gut. Zunft et al. [44] showed already in 1983 that maltitol will be digested and utilized by man, rat, and rabbit. A daily application of 35 g maltitol to humans “did not influence the parameters of well-being, compatibility, and fecal state.”

A study carried out with maltitol indicated that 30 g maltitol in chocolate caused no significant symptoms in young adults, while 40 g caused mild borborygmus and flatus, but no laxation. An increased breath H_2_ response indicated primarily colonic maltitol fermentation [45]. Another study reported that occasional or regular consumption of maltitol was not associated with severe digestive symptoms [46]. In both patterns of maltitol consumption, osmotic diarrhea frequency was higher but appeared only for very high doses of maltitol (about 90 g); maltitol did not lead to intestinal flora adaptation after a 9-day period of consumption. In another experiment, a 45 g dose of maltitol caused transitory osmotic diarrhea in 29 of 34 subjects (85.3%) [11]. The symptoms could be suppressed by simultaneous ingestion of partially hydrolyzed guar gum which consists of the ground endosperm of guar (a legume) seeds. The gum, which swells and disperses in water, contains a mannose- and galactose-based polysaccharide, guaran.

It may be of interest that maltitol has been shown to protect against dimethylhydrazine-induced tumours in rat caecum and proximal colon. This may result from butyric acid formation [47]. Another possible benefit is the maltitol-associated promotion of calcium absorption and advantageous bone effects in rat models [48, 49], a reaction that has also been observed with xylitol [50]. In the past, some countries, including Canada, Australia, New Zealand, Mexico, and Norway, have required manufacturers to include warning labels on packages of maltitol-containing comestibles, since “excessive consumption may have laxative effects.” The FDA has regarded maltitol as a GRAS substance (generally recognized as safe) with a warning about its cathartic potential when consumed at levels above 100 g per day (in adults).

d-Glucitol is also a hydrolysis product of isomalt (molar mass 344.31), which is an equimolar mixture of *α*-d-glycopyranosyl-1-6-d-glucitol and *α*-d-glucopyranosyl-1,6-d-mannitol. The intact portion of isomalt and the unabsorbed d-glucitol and d-mannitol molecules eventually reach the lower parts of the gut where they serve as substrates for bacterial formation of volatile fatty acids. Isomalt may not be consumed by adults in quantities larger than about 50 g per day; flatulence and diarrhea may occur. For children, 25 g per day may represent a practical upper limit. Isomalt represents those disaccharide sugar alcohols that are treated by the human body as “dietary fiber” and not as a regular disaccharide. Consequently, isomalt can pass through the bowel partly undigested; part of it is hydrolyzed in the small intestine. Habitual consumption of isomalt may lead to partial adaptation, which suggests decreased occurrence of gastrointestinal changes. Isomalt could be used as an alternative to lactulose for colonic delivery system utilizing the principles of a unique colon-specific delivery technique called CODES [51]. Since d-glucitol has been associated with greater colonic fermentation compared with isomalt [52], its formation from the latter should be considered.

Lactitol [4-*O*-(*β*-d-galactopyranosyl)-d-glucose; molar mass 344.31] passes through the small intestine almost completely unabsorbed and is subject to microbial fermentation in the distal parts of the gut. Lactitol can cause flatulence and osmotic diarrhea in some individuals, since most subjects lack the necessary *β*-galactosidase enzyme in the upper gastrointestinal tract. After reaching the large intestine, the lactitol molecules can pull water into the gut lumen by simple osmosis. True loading tests with lactitol are limited. In a human study, consumption of 5 g of lactitol per day resulted in no gastrointestinal distress, while 10 g per day did cause some changes [53]. Compare also with Natah et al. [54], whose study subjects reported no abdominal pain after ingesting lactitol.

In conclusion concerning disaccharide sugar alcohols, excessive consumption of maltitol and isomalt can cause significant osmotic diarrhea and flatulence. True gastrointestinal loading tests on lactitol should be repeated. The amount of disaccharide polyols present in chewing gum is too low to cause any gastrointestinal effects in most subjects.

## 5. Involvement of the Raffinose-Family Galactooligosaccharides (GOSs)

The GOSs are not sugar alcohols but may occasion similar gastrointestinal disturbances as the latter. Historically, the GOS group of carbohydrates deserve attention in this context because of the presence of GOS in some polyol-containing manufactured foods. Indeed, field experience suggests that consumers frequently misjudge the causative food agent when simultaneously consuming raffinose-based food of a leguminous nature and sugar alcohol-containing confectionaries or medicines. Therefore, the role of GOS will be concisely discussed here. Legumes, rich in GOSs, normally contain only insignificant quantities of sugar alcohols.

Oligosaccharides began to receive more attention as a result of the growing interest in bringing new sources of protein into the food system, including soybeans, which contain these sugars. Oligosaccharides are not digested because the human alimentary canal does not produce the necessary enzyme, *α*-galactosidase. Nor are oligosaccharides resorbed by the intestinal wall, owing to their high molecular weight. Consequently, they come in contact with bacteria that inhabit the lower parts of the intestine. The bacteria are able to utilize the raffinose-family oligosaccharides with subsequent formation of flatus [55]. These oligosaccharides may also promote the growth of bifidobacteria in the human intestine and cause diarrhea when consumed in excess of a particular quantity [56].

The molecular weight of oligosaccharides has an influence on flatus formation. These compounds include stachyose (molar mass 666.58), a tetraholoside, and verbascose (828.72, a pentaholoside). A holoside is a glycoside that yields only glycoses on hydrolysis. Both have marked effects as flatus formers. Raffinose (or melitose; 504.42), a triholoside, that is,* O*-*α*-d-galactopyranosyl-(1→6)-*O*-*α*-d-glucopyranosyl-(1→2)-*β*-d-fructofuanoside, normally has a less significant effect. The objective of emphasizing the role of ordinary leguminous plants as a source of flatus (and diarrhea) is to underline the role of regular human food as another common source of gastrointestinal discomfort.

Flatulence is an old problem; the first scientific reports dealing with it were published early in the last century. Even a slight increase in pressure in rectal gas may lead to symptoms of discomfort. Researchers discovered about fifty years ago that some GOSs play a part in flatus formation. Flatus is often accompanied by a lowering of the pH. The lowered pH may in turn affect the metabolism of other substances [57, 58].

Microbial fermentations of GOS in the large intestine are responsible for flatus components such as hydrogen, methane, and carbon dioxide. Oxygen and nitrogen may also be present and originate from swallowed air. Significant, positive correlations were discovered between hydrogen production and the following chemical components that are present in various pea varieties: stachyose and raffinose and various glucans and pentosans. A study in patients with ileostomies showed that 88% of raffinose passed unabsorbed through the small intestine; in the same study, 74% of d-mannitol and 100% of lactulose passed unabsorbed [57–59].

## 6. Main Features of Xylitol Metabolism in Humans

Glucose and galactose which are common dietary carbohydrates can be concentrated against a tenfold gradient by an active transport mechanism that assures their early absorption in the intestinal tract [6, 13, 19, 20, 60, 61]. The question is of a facilitated transport mechanism. In the case of xylitol and d-glucitol, however, there is no evidence of such transport mechanisms [3, 60–66]. As mentioned above, their absorption takes place based on free diffusion, or, if an active transport system exists, it has only a low affinity. The driving force behind free diffusion is the concentration difference for the substance in question [65–67]. Another factor limiting diffusion is the pore size [68]. The diameter of hydrophilic pores may range considerably from less than one nanometer to between 0.3 nm and 0.6 nm, but the structures may not assume the shape of pores but, rather, tunnel-like channels. Although the molecular weights of xylitol (152.1) and d-glucitol (182.2) differ by only about 20%, this difference is significant in the borderline range of free diffusion. The symmetrical configuration of the xylitol molecule may facilitate a single-file diffusion of the molecule through tunnels.

**Figure 1:**
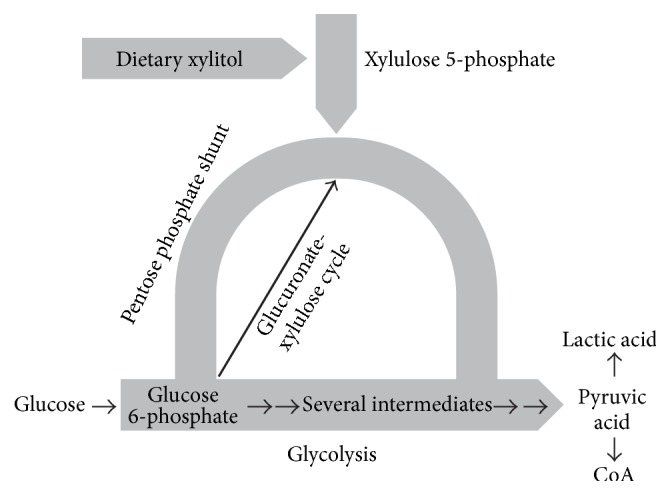
Relationship between the metabolism of xylitol and glycolysis in humans. The scheme describes the metabolism of dietary xylitol in broad outline only. The body receives energy from glycolysis (the thick horizontal arrow). The first intermediate of glycolysis is glucose 6-phosphate which forms an important link between glycolysis and another metabolic pathway, called the pentose phosphate shunt, or pentose phosphate cycle (curved arrow). The thinner black arrow represents the glucuronate-xylulose cycle of Touster. The differences in the thickness of the arrows reflect the relative portion of these three pathways in the overall metabolism. Although the significance of the Touster cycle is minor from the energetic point of view, it is nevertheless absolutely necessary for body functions. Pyruvic acid which may be regarded as the end product of glycolysis can be further metabolized in two ways: reduction to lactic acid under conditions of limited oxygen supply, or becoming a part of coenzyme A when the oxygen supply is sufficient. The scheme shows how xylitol can contribute to the overall energy metabolism of the body. The original scheme of Bässler [67] was modified and completed by the present author.

The greatest portion of absorbed xylitol is metabolized in the liver, although kidneys and other tissues are also sites of xylitol metabolism [19, 54]. Red blood cells metabolize xylitol readily. Most xylitol is metabolized by a pathway involving normal, physiologic enzyme-catalyzed steps of the pentose phosphate pathway. This pathway is a portion of the glucuronate-xylulose cycle, also called Touster's cycle that was introduced already in the 1950s and 1960s [60, 62–66]. It has been difficult to visualize in practical terms the link between this cycle and the better-known glycolysis. It is possible that the German researcher Bässler [67] succeeded in outlining this link graphically (Figure 1). The identity of the enzymes involved in this cycle and the overall metabolism of xylitol in human tissues was established by the mid-1980s, when the United States FDA released its expert opinion on the safety of xylitol and lactose. This resolution (*vide infra*) is still in effect and forms the basis for the FDA's most recent safety statements regarding xylitol and also for the scientific opinion of the Joint Expert Committee of the World Health Organization (WHO) and the Food and Agriculture Organisation (JECFA) resolutions concerning the safety of xylitol.

## 7. True Loading Tests of Xylitol in Humans: A Historical Perspective

Few research papers have reported on gastrointestinal changes during xylitol consumption. This partly results from the nonexistence of such changes in clinical trials aimed at investigating oral biologic and dental effects of xylitol. In most stomatologic studies, xylitol consumption levels have been relatively small, and, consequently, the researchers did not need to focus on possible side effects of xylitol consumption. The scantiness of such reports is unfortunate, since the next generation of consumers, health-care authorities, and medical and dental practitioners has retroactively started to ask for hard data on the relationship between the consumption of xylitol and bowel movements, flatulence, meteorism, and other bowel reactions.

Observations on the occurrence of diarrhea in studies involving consumption of xylitol and other dietary alditols will be reviewed below, as reported by the authors of those studies. The individual studies are summarized instead of showing study details in the form of tables. This results from the publication of several early studies in difficult-to-locate journals, which have not provided abstracts of papers. Since these studies represent real-life situations, their review enables present readers to obtain direct information on the studies involved, with practical instructions regarding dosage levels of alditols for patient counselling purposes.

By the mid-1970s, various medical and dental benefits of xylitol were already known. Considerable experience had become available since the 1960s from the former Soviet Union, where the metabolism and uses of xylitol for nutritive and medical purposes had become a favored research topic. The Soviet researchers were not aware of the dental effects of xylitol until the publication of the Finnish Turku Sugar Studies in 1975 [20]. This study prompted Galiullin [60] to undertake a two-year xylitol trial in the state of Kazan. His results were in line with those of the Turku study (*vide infra*). Some Russian-language medical articles have been difficult to access, but a valuable contribution to this xylitol literature was made by Dr. Nesterin from the Moscow Nutrition Institute. He wrote a comprehensive historic review of the Soviet investigations into the general medical effects of xylitol, including its toxicity, influence on bodily functions in* diabetes mellitus*, disorders of the hepatobiliary system, and other medical conditions. This Russian-language article was translated into English and appeared in 1980 in a German scientific journal [13]. Although the article focused on diabetes and disturbances of the liver and gallbladder system, observations on gastrointestinal effects of xylitol were also made. Nesterin also described a large number of animal experiments. The direct quotes below are examples from the translation.

Nesterin's review showed that the Soviet medical authorities recommended xylitol in the treatment of various medical conditions. Gastroenterological statements indicated that “good tolerance to xylitol was noted in the treatment of children who received 20–35 g xylitol for 4 weeks.” Similar conclusions were made after diabetic children had received 40 g of xylitol daily for one month. In a study carried out at the USSR Academy of Sciences Central Hospital, 55 adult diabetic patients received 30–40 g of xylitol daily for one year. The researchers noted no side effects; “laxative effects never occurred,” while disorders of carbohydrate metabolism disappeared “and the patients felt better.” As a result of these observations, Soviet physicians started to prescribe xylitol to patients suffering from constipation. A common dose was 50–60 g of xylitol which was “consumed well; no pathological symptoms occurred, while the bolus structure normalized (without diarrhoea).” The Soviet researches also stated that female patients, aged 40 to 60 years, and who had liver and gallbladder problems, benefited from a 4-week xylitol program (30 g per day); “dispepsia [*sic*] and painfulness during palpation vanished.” “Side-effects—such as meteorism and watery stools—occurred seldom” [13].

In the mid-1970s, the present author received a personal report from Dr. M. V. Milishnikova who then worked at the Department of Propedeutics of Internal Diseases of Astrachan Medical Institute. Her report represented an account of medical studies entitled “Xylite in Ration of Patients with* Diabetes Mellitus*” [*sic*]. Related to osmotic diarrhea, the following is an excerpt, in direct quotation, from the report: “Twenty-one 41- to 70-year-old diabetic patients received 40 g of xylitol in 200 mL of water per day before a meal. No side effects were observed.” The study focused on the glycemic curve and on the extent of glycosuria, both of which remained within the normal physiologic range. Dr. Milishnikova further stated that “administration of xylitol improved these patients' feeling, and had a favorable effect on bile secretion and emptying of intestine” [*sic*]. Her patients also included diabetic subjects who had frequent pain in the right hypochondrium and suffered from constipation. Following the “xylite treatment (40 g daily), these symptoms disappeared.” She added, however, that the improvement in carbohydrate metabolism was not observed in all patients. It is also possible that the patients' meals contained water-absorbent dietary fibers which may have alleviated gastrointestinal responses.

In another experiment 41- to 50-year-old diabetic patients received 40 g of xylitol divided into 2 or 3 portions during one day. “No side effects such as epigastric pain, nausea, vomiting, and diarrhoea were observed.” The metabolic parameters were normal (a favorable influence on bile secretion was noted). In other experiments a general improvement in the diabetic state of a large number of diabetic subjects was observed. An expected observation was the relief of constipation some subjects suffered. The Soviet physicians concluded that “xylite can be used in diet for patients with* diabetes mellitus.*” Some of these results were published in Russian already in 1967 by I. V. Domareva (in* Vopr. Pitanija*, No. 3, p. 46) and in the same year by M. S. Marshak and I. S. Savoshtshenko (in* Med. Gazeta*, No. 64), as reported by Milishnikova.

Coinciding with the publication of the above Soviet experiences in the German medical journal, several research groups in Germany got engrossed in detailed gastrointestinal studies with xylitol. Research teams in other countries followed suit. The following twenty reports provide quantitative information on osmotic diarrhea associated with the consumption of xylitol by human subjects.

*(1) Three-Week Feeding Study*. Dubach et al. [3] tested a group of 19 subjects of both sexes, aged 21–27 years. The subjects were given xylitol for 21 days in the form of “compressed material” and in jam, increasing the doses up to a maximum of 75 g per day. Intolerance was not observed. After one month the subjects received 40 g xylitol in one single dose without any signs of intolerance. This dose could be increased to a maximum of 220 g/day. At this level, aversion to sweets was noted. Body weight, fasting blood sugar values, and stool consistency remained uninfluenced. Diarrhea first developed at 130 g/day, but, according to the authors, this resulted mainly from poor distribution of single doses. In another experiment, tolerance for xylitol and d-glucitol was compared to levels of up to 75 g per day for up to two weeks. Twenty-one subjects out of 26 preferred xylitol over d-glucitol; meteorism and flatus were more common with d-glucitol at the same dose. The authors concluded that “there were no significant adverse effects with xylitol except for loose stools which could be controlled by appropriate dosing schedule.” 

*(2) Effect of Increasing Dosage*. Asano et al. [61] demonstrated by modern absorptive gastroenterological techniques that, in adult men, xylitol absorption decreases with increasing dosage, being 90% absorbed at 5 g in a single dose, 76% at 15 g, and 66% at 30 g xylitol in a single dose. Up to 30 g of xylitol was found “to be well absorbed by human subjects and to have no adverse effect judging laboratory tests and symptoms.” Asano et al., therefore, showed no incidences of laxation in dosing his adult subjects at a level of 30 g of xylitol as a single bolus. The authors concluded that d-glucitol at a single dose of only 15–30 g leads to diarrhea in young subjects whereas approximately twice this quantity of xylitol (25–40 g) would be required for a similar effect [61]. Förster [68] reevaluated these studies in detail.

*(3) Effect of 120 g Doses.* Amador and Eisenstein adapted five persons with increments of 30 g of xylitol per day in three individual doses at three-day intervals up to 120 g per day. The authors concluded that, overall, there was “virtually no gastrointestinal stress at less than 90 g/day.” It should be noted that the subjects who showed diarrhea at 90 g per day weighed only about 40 kg and that the tolerance was greater than 90 g to 100 g of xylitol per day for an adult, 100 g being the highest level tested with adaptation. This study was described in detail by Brin and Miller in 1974 [69]. 

*(4) Two-Year Feeding Trial.* A long-term feeding trial on xylitol was carried out in 1972–1974 in Turku, Finland [4, 20]. Three groups of volunteers, totalling 125, lived for two years on strict diets so that comparisons could be made with regard to the sweeteners tested: sucrose (S), fructose (F), and xylitol (X). These diets were given to the subjects free of charge from the institute carrying out the research. A total of about twelve food manufacturing enterprises participated in providing a wide variety of food items for the subjects [4, 20]. This study constitutes perhaps the most compelling and detailed evidence so far on the effects of long-term uninterrupted consumption of a sugar alcohol in humans in a situation where the average daily quantities of the substance amounted to about 67 g per day. Consequently, since this study remains the only long-term true feeding trial with any sugar alcohol conducted in humans and since the above publication (a 1975 supplement to* Acta Odontologica Scandinavica*) has not been readily available, this research is summarized below.

The sizes of the test groups were S, 35; F, 38; X, 52. The average daily amount of the sweeteners consumed in a varied assortment of food was S, 73 g; F, 70 g; X, 67 g. (The calculated consumption value for sucrose was most likely somewhat higher, since subjects were known to consume food obtained from other sources.) In this study, the highest daily doses of xylitol were 200 to 400 g. The subjects were continuously monitored by medical research teams. The study showed that the consumption of xylitol and fructose was associated with osmotic diarrhea, flatulence, and gastric distress.

**Figure 2:**
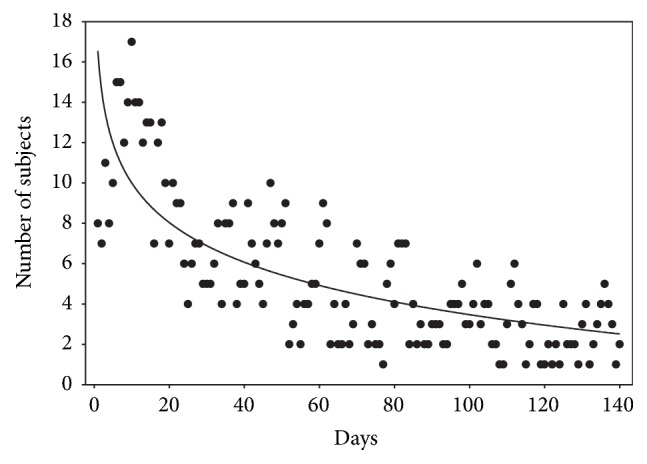
Gradual lessening of osmotic diarrhea and flatulence in human subjects who consumed on the average 67 g of xylitol daily for two years. The results are here shown for the first 140-day period. The ordinate gives the number of subjects complaining even about slight diarrhea or increased defecation frequency on each test day. The initial peaks of consumption were found to result from the interest of the subjects to get acquainted with the new dietary regimen. Modified from [4].

The ability of xylitol to produce gastrointestinal disturbances was found to depend on individual physiological responses in each volunteer. In many cases, subjects reported no symptoms even though high amounts of xylitol were consumed. All pregnancies and deliveries in the xylitol group were normal. The overview of the trial [20] concluded that “the osmotic diarrhea that occurred in a number of subjects after heavy peroral xylitol loading gradually disappeared as a phenomenon of adaptation took place” (Figure 2). The illustration shows the number of subjects who reported loose stools during the first 140 days of the trial. As expected, several subjects experienced loose stools during the first weeks. After the first 140 days, the frequency of symptoms continued almost unchanged for the rest of the study. Consequently, during the last 590 days of the feeding trial, the occurrence of diarrhea in the xylitol group was nearly of the same frequency as in the sucrose and fructose groups; that is, the reports about diarrhea decreased to about one-quarter compared to the first weeks. The total number of intakes of xylitol-containing food items was 129,000 over the course of the two-year trial, or about 110 reported intakes of xylitol food per subject and per month. A total of 35 subjects in the xylitol group were considered as having consumed exceptionally high quantities of xylitol. Within this 35-subject group, the overall number of days with an intake of 100–149 g of xylitol was 1,416 over two years. In these subgroups, the overall numbers of days with an intake of 150–199 g and >200 g were 230 and 64, respectively.

After the adaptation phase of about three weeks, it was noted that several subjects had not reported diarrhea-like conditions even though they were deliberately attempting to cause laxative effects by consuming 60 g of granulated xylitol as a single dose. Out of the initial number of subjects (57) who started the xylitol regimen, five discontinued the program as a result of study fatigue (2 subjects), poor compliance (one subject), employment reasons (one subject), and reported persistent diarrhea (one subject). The final medical reports (including gastrointestinal information) were thus available from 52 xylitol-consuming subjects. This information was obtained by means of written diaries and was considered somewhat subjective. It is possible that the levelling off of the regression curve in Figure 2 resulted in part from subjects gradually learning to use xylitol food in moderation. However, a true adaptation in the intestinal flora and liver was most likely also involved. The researchers concluded that “xylitol was well tolerated by the majority of the subjects.”

*(5) Two-Year Soviet Union Study.* As mentioned above, following the completion of the Turku Sugar Studies [4, 20], the first confirmatory clinical evidence of the caries-limiting qualities of xylitol was obtained from the study published in 1981 by Galiullin [60]. In this two-year trial, 8- to 14-year-old subjects received 30 g of xylitol daily in the form of candies. The comparison group received 60 g of sucrose in the form of similar candies. The objective was to replace one-half of daily consumption of sweets in the xylitol group. In addition to registering of dental caries outcomes (which showed xylitol to reduce caries incidence by about 70% compared with sucrose), the study investigated several anthropometric, pulmonary, otolaryngeal, rheumatologic, endocrinologic, and metabolic parameters of the subjects. The children's comprehensive physical check-ups revealed no differences between the xylitol and the control groups, apart from significantly lower caries incidence in the former. The groups also did not differ with regard to bowel movement recordings. 

*(6) University of Texas Study.* A study entitled “Oral Xylitol in Humans” was published by Wang et al. [70]. The study was carried out at the University of Texas System Cancer Center in Houston. Seventeen adult subjects of both sexes received xylitol enterally so that the xylitol level was gradually increased from 3 × 10 g per day to 2 × 50 g per day over a 14-day period, with the final dose maintained for 3 days. The study investigated a total of 56 clinical-chemical parameters. Severe diarrhea was observed in one male subject when the xylitol dose was 3 × 20 g per day. Milder diarrhea and flatulence were reported in all subjects. Adaptation to xylitol was observed. The authors concluded that “the adult human can tolerate substantial amounts of daily xylitol.”

*(7) Reexamination of the Turku Sugar Study Subjects.* The general health of the participants in the above-mentioned Turku Sugar Studies [4, 20] was reexamined four years following the final xylitol feeding [71, 72]. These reexaminations included a special comparison of metabolic tolerance test of nine “xylitol chronics,” that is, human volunteers who had used xylitol regularly for 4.4–5.3 years (the first two years in the capacity of participants in the original two-year feeding trial). In this tolerance test, the subjects consumed, over 7 days, 70–100 g of sucrose per day with the basal diet (as in the case of the study of Förster et al.;* vide infra*), followed by the consumption of 70–100 g of xylitol per day in the basal diet for 14 days, and similar consumption of xylitol in normal diet for 7 days. This basal diet (formula diet) did not contain fiber and thus lacked the water-binding capacity of normal food. The subjects were investigated using versatile clinical, anthropometric, ophthalmological, and metabolic tests. The xylitol loading tests were not found to result in any abnormal metabolic reactions. As expected, the sudden increase in the level of xylitol consumption from those to which the subjects were accustomed resulted in osmotic diarrhea in some subjects. These symptoms disappeared in most cases in 3 to 4 days. No significant diarrhea was reported by subjects who consumed normal diet plus xylitol. Four instances of diarrhea (in two subjects) and six instances of flatus (in three subjects) were recorded during the basal sucrose diet and normal diet periods (without xylitol). Upon completing this review, all nine “xylitol chronics” are alive, the oldest ones being nearly eighty years old. Four of them have continued uninterrupted daily consumption of xylitol over 44 years. 

*(8) 55-Day Study in Children*. Åkerblom et al. [7] studied the tolerance of increasing amounts of dietary xylitol in healthy children aged 7–16 years. Xylitol was incorporated into the diet in the form of chocolate, chewing gum, wafers, crystalline xylitol, meringue candies, yoghurt, and ice cream. The daily dose was increased from 10 to 25, 45, 65, and 80 g (in successive 10-day increments) and finally decreased to 65 g for 5 days. Gastrointestinal side effects were recorded daily during the 55-day xylitol consumption, as well as during xylitol-free periods before and after the trial. Flatulence was the most common side effect occurring infrequently in about half of the subjects during the 45 g/day intake of xylitol and in the majority of the children at higher doses. During the latter periods of high-level xylitol administration, an obvious adaptation to the substance was observed. Transient diarrhea (but no increase in the number of stools) occurred in four children at 65 g/day xylitol consumption and in one child at 80 g/day. The authors concluded that “a reasonable consumption of xylitol in the form of chewing gums and small candies or confections is harmless for children, and can be recommended when this would replace consumption of similar confections sweetened with sucrose or other cariogenic sweeteners.”

*(9) German Study in Healthy Adults.* Förster et al. [73] carried out a study on 12 healthy volunteers who consumed a standardized basal diet consecutively supplemented with either sucrose (6 days, 60–100 g/day) or xylitol (18 days, 40–100 g/day). With the exception of a few cases of diarrhea only at the start of the xylitol regimen, no other clinical signs indicated treatment-related side effects. This finding was considered remarkable, since the liquid nature of the formula diet consumed is devoid of fiber (and hence lacks water-binding capacity) and the subjects investigated had not been previously exposed to xylitol. (In the previous xylitol loading test of a similar nature [71, 72], subjects were partially adapted to xylitol.) The subjects were allowed to reduce somewhat the xylitol dosage until diarrhea subsided, although, in cases where diarrhea occurred or persisted, the achieved levels of xylitol nevertheless corresponded approximately to the targeted level of up to 100 g/day. “This provided further evidence that the gastrointestinal tolerance of the subjects was good” [73].

In an earlier paper, Förster [68] referred to older German experiments which indicated that xylitol was well tolerated by children and diabetic subjects. For example, in a study carried out by Mellinghoff already in 1960 (published in 1961), xylitol was used as a substitute for sugar with diabetics. Using low dosages (10 g per day), there were no symptoms of diarrhea. Only at higher dosages (60 g in tea), did cases of diarrhea occur. In another experiment of his own, Förster found that 100 g of xylitol was tolerated “without much difficulty” by six volunteers over a period of ten days [68]. Förster found no adverse gastrointestinal effects during administration of 30 g of xylitol over a period of four weeks to diabetic children. Förster mentions in his paper also a study by Mertz et al., who observed no symptoms after their subjects had consumed 50 g xylitol, and a study with diabetic children who received 30 g xylitol per day over a period of four weeks. Only one child withdrew prematurely from the sequence of experiments on account of diarrhea [68]. 

*(10) Chronic Xylitol Users.* Diarrhea-associated data of 11 subjects, who had habitually used xylitol for 3.2 to 4.5 years, was published in 1977 [74]. Four of the subjects had also participated in the above-mentioned xylitol loading test [72]. The group of 11 included three children who had used xylitol for most of their lives. Their ages at the commencement of the program were 1.4, 2.6, and 12.1 years. Six adult subjects in this group had also participated in the two-year Turku Sugar Studies (1972–1974) involving, on the average, 67 g intake of xylitol per day in the form of versatile xylitol products [4, 20, 71, 72]. Following the termination of the feeding study, that is, during the next 2.5 years, the six subjects consumed xylitol daily mostly in the form of chewing gum, troches, and chocolate, at consumption levels ranging from 1.4 kg per year to 11 kg per year. Two additional adults in the 11-subject group had used a total of 58 kg and 24.8 kg of xylitol, respectively, during 1972–1974, and 19.0 kg and 22 kg, respectively, over the next 2.5 years (the 2.5-year figures resulted mostly from the use of confectioneries). Detailed paper diary and questionnaire performances showed that none of the subjects reported diarrhea during the entire study period (the children's data were based on parental monitoring). Absence of gastrointestinal disturbances in the two youngest children was noticeable. Their average daily frequency of xylitol intake varied from 3 to 7 during their 3.3- or 4.5-year participation. 

*(11) Effect on Gastric Inhibitory Polypeptide*. The group of Salminen [5] studied six healthy volunteers, aged 26–36 years, who were unaccustomed to xylitol. The subjects received a single 30 g xylitol dose in 200 mL water after a 12 h fast. Two subjects experienced transient diarrhea and one complained of flatulence. An important observation was that this xylitol administration had no effect on the concentration of gastric inhibitory polypeptide or insulin in plasma. In another study, an aqueous solution of xylitol (25 g/50 mL) was used to study gastric emptying (to wash down a scrambled-egg meal). After ingestion of xylitol, gastric emptying was markedly prolonged. Xylitol decreased food intake, causing the authors to suggest a role for xylitol as a potentially important agent in dietary control [75]. Salminen et al. stated in a later study [6] that two of six healthy 22- to 35-year-old volunteers reported sudden transient diarrhea 2-3 h after xylitol consumption and that all six had softer stools and increased stool frequency after xylitol intake. In this case the subjects received a 200 mL drink containing 30 g xylitol or 30 g glucose. 

*(12) WHO Study.* In a collaborative Hungarian World Health Organization xylitol field study carried out during early 1980s, institutionalized 6- to 11-year-old hearing- and sight-impaired children or orphans (*n* = 278) received 14–20 g of xylitol daily over a period of three years. During the entire course of the study, no problems were encountered with regard to the reported frequency of laxation or possibly associated abdominal discomfort [76]. 

*(13) Oral Xylitol in American Adults*. Twelve healthy adult subjects were given xylitol in incrementally increasing daily doses from 30 g in three doses to 100 g in two doses along with a regulated diet [77]. All subjects experienced dose-dependent diarrhea. One of the subjects was intolerant of doses greater than 20 g, while 11 subjects tolerated daily doses of up to 100 g. Adaptation was observed in most subjects. The authors concluded that “oral xylitol in combination with normal American diet imposes no side effects other than gastrointestinal intolerance as those observed in West Germany and Scandinavian countries.”

*(14) Metabolic Responses to Xylitol and Lactitol.* Eight healthy, nonobese male subjects with a mean age of 25 ± 1 years were studied after 10 to 12 h fast. The subjects ingested, in 250 mL water, either 25 g glucose, 25 g xylitol, or 26.25 g lactitol monohydrate within 2-3 min. None of the subjects had abdominal pain or diarrhea during the study [54]. 

*(15) Seattle Studies*. Lam's group at the University of Washington used xylitol-containing foods in xylitol feeding studies in young children aged 3 to 6 years [78]. The foods included popsicles, puddings, gum drops, gelatin dessert, cookies, and popcorn. This experiment was not a loading test but measured children's acceptance of xylitol-based foods; the amount of xylitol presented to the children on a tray of xylitol foods was up to 2.4 g per episode. These snack foods were generally well tolerated by children. In another experiment xylitol-containing milk was well accepted by 4- to 7-year-old children [79]. 

*(16) South Korea Study.* In a kindergarten study carried out in South Korea in 2002-2003, 123 5-year-old children were divided into three groups of equal size. Two of the groups received, in the form of chewing gum, 4.5 to 5.0 g of xylitol or d-glucitol, respectively, daily for six months, with one group serving as a comparison [80]. None of the subjects had gastrointestinal problems, as reported by kindergarten personnel and parents. The children regarded the use of chewing gum as a pleasurable experience. 

*(17) Comparison between Erythritol and Xylitol.* Sixty-four adult subjects completed a study where the gastrointestinal responses to single oral bolus doses of erythritol and xylitol (20, 35, or 50 g) were investigated [8]. These subjects can be regarded as unaccustomed to the polyols tested. Compared with a 45 g sucrose dose, 50 g xylitol in water significantly increased the number of subjects reporting nausea, bloating, borborygmus, colic, watery feces, and total bowel movement frequency. The 35 g xylitol dose increased bowel movement frequency of passing watery feces, while 50 g erythritol significantly increased the number of subjects reporting nausea and borborygmus. Lower doses of 20 and 35 g erythritol did not provoke a significant increase in gastrointestinal symptoms. 

*(18) Infant Study*. Six- to 36-month-old infants received xylitol in 5 g doses thrice per day or 7.5 g once a day in the form of an aqueous solution for three months (to assess the effect of xylitol on* otitis media*). A 5% d-glucitol solution was used as a control. Gastrointestinal complaints, excessive gas, diarrhea, and vomiting were monitored. The authors reported that “the infants tolerated the oral xylitol solution well” [81]. 

*(19) Japanese Study in Adult Subjects.* The noneffective dosage of three sugar alcohols not causing transitory diarrhea was investigated in 27 male and 28 female subjects in a Japanese study [82]. The test substances (10 to 50 g/150 mL water) were consumed 2-3 h after meal. The noneffective dose level of xylitol was 0.37 g/kg body weight for males and 0.42 g for females. The corresponding values for lactitol were about 20% to 33% smaller, while erythritol was better tolerated: 0.46 g/kg body weight for males and 0.68 g/kg for females. 

*(20) Japanese Study in Preschoolers*. Xylitol chewing gum was given to 3-4-year-old preschoolers in a Japanese study [83]. This study was chosen for the present piece to represent another attempt at monitoring the occurrence of gastrointestinal side effects in a regular chewing gum study in young children. The authors managed to monitor the occurrence of osmotic diarrhea in the children with the aid of parental participation. The children were supposed to chew one gum pellet 4 times/day for 3 months, that is, 4 × 90 (approximate number of test days) = 360 pellets* in toto*. The required daily consumption of xylitol was planned to amount to 5.32 g per day. The percentage of the children who experienced diarrhea during the xylitol consumption period was 11% (8 subjects out of 76). Interestingly, 24% of children (11 out of 45) who did not consume xylitol gum “well” (i.e., their cumulative gum consumption was fewer than 100 pieces in 3 months) experienced diarrhea, a proportion larger than among the “well-consumed” children (11%).

## 8. General Conclusions on Loading Studies with Xylitol

Following the clinical and laboratory xylitol studies in humans and experimental animals completed by the mid-1980s, the FDA commissioned the Life Sciences Research Office (LSRO) of the Federation of American Societies for Experimental Biology (FASEB) to review and evaluate the available biomedical information on sugar alcohols and lactose. FASEB and LSRO provide scientific assessments of topics in the biomedical sciences. Reports are based on comprehensive literature reviews and the scientific opinions of knowledgeable investigators engaged in work in relevant areas of biology and medicine. Health-care authorities around the world frequently base their opinions on these regulatory and scientific bodies of the United States, that is, FDA, FASEB, and LSRO. Inclusion of lactose in the scientific survey of sugar alcohol research can be regarded as a wise tactical decision. After the publication in 1986 of the final scientific opinion, entitled “Health Aspects of Sugar Alcohols and Lactose” [84], there has been no need to examine the safety of xylitol and lactose further. Authorities in various countries have, when necessary, referred to this FDA resolution. New information regarding the absorption and metabolism of xylitol and other sugar alcohols has become available after the publication of the joint FDA-LSRO resolution. The new information, some of which was detailed in cases (1)–(20) above, has confirmed the historic knowledge [84] and is in congruence with the FDA-LSRO resolution regarding the occurrence of gastrointestinal effects associated with the consumption of xylitol and other sugar alcohols. Ten years after the above joint FDA-LSRO resolution, the FDA announced its “Final Rule” regarding the use of sugar alcohols in the nonpromotion of dental caries, referring to the GRAS-listed status of xylitol [85]. By the mid-1980s, the advent of erythritol as a dietary sweetener had not yet occurred.

Gastrointestinal side effects normally occur after consumption of excessive doses of slowly absorbed carbohydrates such as lactose and sugar alcohols (apart from erythritol). The severity of symptoms depends on the individual consumer, state of fasting, dose consumed, mode of ingestion, molecular characteristics of the test substance, composition and structure of the other food simultaneously consumed, and existence of any prior period of adaptation [86]. Protection of the consumer from polyol-induced diarrhea can best be achieved by providing appropriate instructions on food label. Accordingly, the Scientific Committee for Foods (SCF) of the European Economic Community (EEC) emphasized already in the late 1980s that ingestion of 20 g d-mannitol and 50 g d-glucitol daily in the form of commercial food products must bear a warning statement indicating that “excess consumption may have a laxative effect.” At that time no corresponding requirement was formulated by EEC-SCF for xylitol, since the general understanding was that xylitol consumption does not normally cause gastrointestinal problems when xylitol is used “for dental purposes, even by diabetic subjects.” Such formulations are now available by the European Union (EU), however, but only concerning authorized EU dental health claims, and not specifically regarding diarrhea [87]. Osmotic diarrhea was touched upon only by stating that “there is a risk of osmotic diarrhea at excessive intakes of polyols. Children younger than 3 years should not use chewing gum (owing to choking hazard).”

The absoluteness of the above-mentioned age limit is understandable in view of the composition of SCF and because relatively few studies in infants have been carried out explicitly from the point of view of gastrointestinal polyol effects. Most likely, some of the SCF members lacked personal, long-term in-family experience in the use of xylitol. Long-term field experience obtained especially within Finnish families, kindergartens, and day-care centers strongly points to the role of families and public institutions in teaching children to use xylitol chewing gum properly as part of lunch programs and oral hygiene practices. Accordingly, a large number of 2- and 3-year-old Finnish children have customarily received xylitol products under parental guidance.

Virtually all energy for the uptake of xylitol from the intestinal lumen is offered by the concentration gradient [19]. If the absorption capacity of xylitol is exceeded, osmotically induced diarrhea may occur. It has been difficult to determine the proportion of orally administered xylitol that is absorbed from the intestinal lumen in each particular set of circumstances. This portion seems to depend, among other things, on whether direct oral intake or consumption in combination with solids is involved. It has nevertheless been established that even when consumed in the most direct form in solution, a significant portion of xylitol will be absorbed, since the associated metabolic effects can only be interpreted in this way [4, 19]. The single dosage of xylitol that is normally tolerated without diarrhea by healthy humans ranges from 10 g to 30 g, although considerable variation may occur between individuals [3, 4, 68]. Some adults can probably tolerate up to more than 200 g xylitol daily, provided the dosage is increased gradually and that such quantities are consumed during the entire day and not as a single bolus [4]. In general, after adaptation, adults will tolerate 20–70 g of xylitol daily without great difficulty [4, 68, 69].

It is necessary to recall that, for “dental purposes” (i.e., for caries limitation), it may not be necessary to consume more than 10–15 g of xylitol daily, provided that this dose is taken in several smaller portions; protection against caries is more effective when xylitol is used in several smaller quantities during the day [1, 4, 20]. Success in caries prevention by xylitol also relies on general oral hygiene and dietary practices; xylitol may not compensate for serious neglect of oral hygiene. The previously recommended 5 to 7 g daily doses of xylitol for caries prevention were based on early xylitol trials. Since some researchers have tested even smaller daily doses, employing caries-resistant study cohorts, too-short intervention periods, simultaneous use of fluoride, and other procedures that have unnecessarily impoverished the intended xylitol program, this author now recommends 10 to 15 g daily xylitol doses for caries prevention. In case of rampant caries and poor oral hygiene, the doses may be even larger. Naturally, several other precautionary steps must also be taken when planning a xylitol-based caries program [1].

The current concept of xylitol as a safe dietary food ingredient is largely based on the above metabolic follow-up and loading tests [7, 71–73] carried out in connection with the Turku Sugar Studies [4, 20] and on the “defensive animal experiments” that led to a joint FDA-FASEB-LSRO release on the safety of xylitol [84]. These safety aspects include gastrointestinal effects of xylitol, such as its slow absorption and potential causing of osmotic diarrhea in situations where recommended upper consumption limits are exceeded by unaccustomed subjects.

Tolerance to xylitol is better when it is consumed as part of regular meals or snacks. Even when consumed in confectionery items, such as pastilles, troches, lozenges, chocolate, and chewing gum, the risk of osmotic diarrhea is not remarkable, since those items are normally used in smaller amounts. Xylitol present in beverages normally causes diarrhea at lower xylitol levels than when present in solid items (this also applies to d-glucitol). Experience from the Turku Sugar Studies [4, 20] suggests that it may not be advisable to use xylitol as a sweetener in soft drinks. Since use of coffee and tea is normally self-restricting, it is possible to use xylitol as a sweetener in coffee and tea without notable gastrointestinal symptoms. Simultaneous consumption of fiber-rich food will lessen the ability of xylitol to cause osmotic diarrhea. Such fibers include cellulose and xylans (a group of so-called hemicelluloses) which impute water-holding properties, resulting in considerable bulking of digesta. Cereals, among other plant-derived foods, are rich in xylans.

Long-term field experience has shown that even health-conscious consumers may be unable to differentiate between mild gastrointestinal effects occasioned by such common dietary items as legumes, lactose, and d-glucitol, if these are consumed together with xylitol. Furthermore, several xylitol-containing confectionery items also contain glucose syrups and maltose syrups or polydextrose—popular bulking agents and sugar replacers which are slowly absorbed and may cause similar effects as polyols. Although polydextrose has been claimed to provide physiological effects similar to those of other fibers and to be better tolerated than most other low digestible carbohydrates (such as polyols), excessive consumption of polydextrose can lead to osmotic diarrhea [88]. Finally, it may always be justifiable to contemplate the possibility that humans may to a certain extent have addled our nutrition by continuous introduction of partly or fully synthetic food ingredients to replace the traditional ones that have constituted our evolutionary environment.

## 9. The Positive Erythritol Response

The four-carbon erythritol is a tetritol that shares many of the functional and physicochemical properties of the sugar alcohol family [1, 15, 89, 90]. Erythritol has gained an increasing number of applications in food manufacturing and in medical and other uses. Owing to the advent of erythritol as a sweetener in foods, attention was directed at the gastrointestinal reactions associated with erythritol consumption. Following extensive safety evaluations (reviewed in [15]), it has been concluded that erythritol is well tolerated in humans and does not cause any toxicologically relevant effects even following ingestion of larger quantities. Tolerance studies [89] confirmed that repeated ingestion of erythritol in amounts of 1 g/kg body weight was well tolerated by humans. No laxation was observed when adults consumed a single bolus of erythritol (in a beverage; 0.7 g/kg body weight) in 15 minutes on an empty stomach. No laxation was observed in 4- to 6-year-old children either. These consumption figures indicate the safety of erythritol use, especially when it has been estimated that the exposure to erythritol via oral health-care products (such as chewing gums and troches) will be very low, that is, approximately 0.1 g/kg body weight per day [90]. The observation that erythritol at doses of up to 0.8 or 1.0 g per kg body weight is well tolerated by the digestive track was demonstrated already by mid-1990s [91, 92] and later corroborated by Jacqz-Aigrain et al. [93]. The human intestinal microflora does not ferment erythritol [94]. Erythritol is normally better tolerated than xylitol by humans.

A study carried out in nondiabetic adults at the Louisiana Technical University showed, however, that a combination of 33.3 g of erythritol and 50 g of fructose (used in equimolar concentrations) increased watery stools and worsened the gastrointestinal tolerance [95]. The authors concluded that coingestion of equimolar concentrations of fructose and erythritol may increase carbohydrate malabsorption; that is, paracellular absorption of erythritol could also enhance paracellular absorption of fructose in healthy adults. The results of Kim et al. [95] and Putkonen et al. [96] suggest that coingestion of equimolar concentrations of fructose and erythritol increases carbohydrate malabsorption. Combinations of erythritol-fructose and erythritol-glucose may also cause untoward effects in dental plaque. Recent studies suggested that erythritol can have utility value in caries prevention [97, 98].

In some experimental animals erythritol may also react different. Guinea pigs were given erythritol with or without the addition of pectin. The endolymphatic volume of the animals was investigated to consider the possibility that erythritol is applicable as a therapeutic agent in Ménière's disease [99]. The feces were muddy in all animals with the uptake of erythritol alone, while muddy or very soft feces were not observed in animals fed a mixture of pectin and erythritol.

## 10. Conclusions and Instructions

- Various gastrointestinal discomforts have been known to humans for thousands of years. Osmotic diarrhea, catharsis, meteorism, flatulence, and borborygmi (borborygmus) are terms that frequently appear in this context.
- Osmotic diarrhea may result from the consumption of too-large doses of dietary sugar alcohols such as xylitol, d-glucitol, d-mannitol, maltitol, lactitol, and isomalt. Also other related substances, such as the GOS and lactose, may cause similar effects. GOS-type substances are normal constituents in the seeds of leguminous plants, such as soya beans and peas.
- Sugar alcohols, along with some oligosaccharides, have also received attention in food and nutrition research owing to their prebiotic properties and other health benefits. IBS and functional constipation serve as examples of common gastrointestinal disorders whose treatment may benefit from the application of sugar alcohols and certain GOSs.
- Osmotic diarrhea occasioned by excessive consumption of these substances is not a disease, but a simple osmotic response to the presence of slowly absorbed carbohydrates in the gut lumen. The presence of these solutes in the lumen will draw water from surrounding tissues.
- The capacity of the common alditols to cause osmotic diarrhea depends on their molar mass, symmetry of the molecule, and, thus, the detailed configuration of the molecule.
- Consumption of erythritol does not normally lead to any gastrointestinal changes, while that of hexitols (d-glucitol and d-mannitol) may cause changes in adults already at 10 to 20 g daily consumption levels. Xylitol is better tolerated, the largest safe doses ranging widely, normally from 20 g to 70 g per day. However, significant variation may occur. Consumption of disaccharide sugar alcohols maltitol, lactitol, and isomalt may also lead to similar gastrointestinal disturbances.
- The quantity of xylitol currently recommended for caries limitation is about 10 g/day or more for adults and about half that for infants older than 3 to 4 years; younger infants have received smaller quantities under parental guidance.
- European Union recommends that daily ingestion of 20 g of d-mannitol and 50 g of d-glucitol in the form of commercial food products should bear a warning statement about possible laxative effects.
- Researchers have not always paid attention to study conditions, such as comparing administration of sugar alcohol in plain water versus as part of regular fiber-containing meals or snacks. For example, tolerance to xylitol present in beverages (such as lemonades, fizzes, and still drinks) normally causes diarrhea at lower xylitol levels than when present in solid food. Use of xylitol in a beverage (apart from as a sweetener in tea of coffee) cannot be recommended.
- Adaptation to tolerate increasing quantities of xylitol has been observed in long-term feeding trials. The adaptive changes take place in the gut flora and possibly by enzyme induction in the liver.
- Xylitol, other alditols, and disaccharide sugar alcohols possess undeniable utility value in dietary and medical applications. Therefore, health-care professionals should be aware of restrictions and recommendations regarding their safe and appropriate use.

## Figures and Tables

**Table 1:** Relationship between the molarities and grams per 100 mL values in aqueous solutions of some dietary sugar alcohols and sucrose.

Sweetener (number of OH groups in the molecule)	Molecular weight (g/mole)	Molarity of a 5% solution	Molarity of a 10% solution	Molarity of a 35% solution
Erythritol (4)	122.1	0.409	0.919	2.866
Xylitol (5)	152.1	0.328	0.657	2.301
Sorbitol (6)	182.2	0.274	0.549	1.921
Mannitol (6)	182.2	0.274	0.549	1.921
Sucrose (8)	343.3	0.146	0.292	1.022

**Table 2:** Maximum bolus doses of some dietary sugar alcohols not causing catharsis. Based on de Cock [15].

Sugar alcohol	Maximum sugar alcohol dose (g/kg body weight)	
	Male	Female
Erythritol	0.66	0.80
Xylitol	0.3	0.3
	0.35–0.4^*∗*^	0.35–0.4^*∗*^
D-Glucitol	0.17	0.24
D-Mannitol	0.3	0.3

^*∗*^Based on the 2-year Turku feeding study in adult subjects accustomed to xylitol [4].
